# TB Incidence in an Adolescent Cohort in South Africa

**DOI:** 10.1371/journal.pone.0059652

**Published:** 2013-03-22

**Authors:** Hassan Mahomed, Rodney Ehrlich, Tony Hawkridge, Mark Hatherill, Lawrence Geiter, Fazlin Kafaar, Deborah Ann Abrahams, Humphrey Mulenga, Michele Tameris, Hennie Geldenhuys, Willem Albert Hanekom, Suzanne Verver, Gregory Dudley Hussey

**Affiliations:** 1 South African Tuberculosis Vaccine Initiative, Institute of Infectious Disease and Molecular Medicine, Cape Town, South Africa; 2 School of Child and Adolescent Health, Cape Town, South Africa; 3 School of Public Health and Family Medicine, Cape Town, South Africa; 4 Vaccines for Africa Initiative, Cape Town, South Africa; 5 University of Cape Town, Cape Town, South Africa; 6 Otsuka Pharmaceutical Development and Commercialization, Inc., Rockville, Maryland, United States of America; 7 KNCV Tuberculosis Foundation, The Hague, The Netherlands and CINIMA, Academic Medical Centre, Amsterdam, The Netherlands; 8 Honorary Research Associate, University of Cape Town, Cape Town, South Africa; Copenhagen University Hospital, Hvidovre, Denmark

## Abstract

**Background:**

Tuberculosis (TB) is a major public health problem globally. Little is known about TB incidence in adolescents who are a proposed target group for new TB vaccines. We conducted a study to determine the TB incidence rates and risk factors for TB disease in a cohort of school-going adolescents in a high TB burden area in South Africa.

**Methods:**

We recruited adolescents aged 12 to 18 years from high schools in Worcester, South Africa. Demographic and clinical information was collected, a tuberculin skin test (TST) performed and blood drawn for a QuantiFERON TB Gold assay at baseline. Screening for TB cases occurred at follow up visits and by surveillance of registers at public sector TB clinics over a period of up to 3.8 years after enrolment.

**Results:**

A total of 6,363 adolescents were enrolled (58% of the school population targeted). During follow up, 67 cases of bacteriologically confirmed TB were detected giving an overall incidence rate of 0.45 per 100 person years (95% confidence interval 0.29–0.72). Black or mixed race, maternal education of primary school or less or unknown, a positive baseline QuantiFERON assay and a positive baseline TST were significant predictors of TB disease on adjusted analysis.

**Conclusion:**

The adolescent TB incidence found in a high burden setting will help TB vaccine developers plan clinical trials in this population. Latent TB infection and low socio-economic status were predictors of TB disease.

## Introduction

Tuberculosis (TB) is a major public health problem globally with 8.8 million cases being diagnosed in 2010 of whom 1.1 million died [1]. Incidence rates start to rise in adolescents and precede a peak in adulthood in developing countries [2]. New TB vaccines are being developed as part of the strategy to combat TB and adolescents are a proposed target for such vaccines [3]. However, relatively little has been published on the incidence of TB in adolescents. Two clinical trials of BCG in adolescents provide limited incidence data [4], [5] and studies on TB in adolescents have focused on the clinical features of TB in this group [6]–[9]. A latent TB infection prevalence of about 50% has been reported for the population described in this report and for another adolescent group from South Africa [10], [11].

Since there is currently no immune correlate of protection against TB, end points based on evidence of tuberculosis disease will be needed for new TB vaccine efficacy trials and these would need to be specific to optimize efficacy assessments [12], [13]. Knowledge of TB incidence rates based on specific clinical disease features will be crucial for planning the size of efficacy trials.

We conducted a study to determine the TB incidence rate and risk factors for TB disease in a cohort of school-going adolescents in a high TB burden area in South Africa. Other publications on this study population include: 1) An examination of risk factors for latent TB infection based on the tuberculin skin test (TST) and QuantiFERON TB Gold (in-tube) (QFT) assay results at enrolment [11] 2) A comparison of the predictive value of baseline TST and QFT for TB disease during follow-up [14] 3) The determination of the predictive value of a QFT conversion for TB disease in a subset of participants who underwent extended follow up [15] and 4) The measurement of the prevalence of TB disease at enrolment and the value of certain screening tests for detecting TB (manuscript under review).

## Materials and Methods

### Study Setting

The study took place in the town of Worcester and surrounding villages, approximately 100 kilometres from Cape Town, South Africa. The total population of the municipal area from which the adolescents were drawn was estimated as 146,101 by the Department of Health in 2005, the year when recruitment started. According to the national Census of 2001, there were 21,056 adolescents aged 12–18 (14% of the total population) in the municipal area targeted and 83% of these adolescents were attending schools. The study area was a subset of the municipal area and consisted of a major town and two villages within this area. Based on the Census 2001 population data for the municipal area and high school attendance data of 10,492 in the study area, we estimated that there were 12,641 adolescents aged 12–18 years in the study area in total.

### Study Participants

All adolescents aged 12–18 years attending all 11 publicly funded high schools in the study area were approached to participate. The very few small private schools in the study area were not approached.

### Study Procedures

At enrolment, demographic, socio-economic and clinical information were collected through interview of parents and the participating adolescent [11]. Blood was taken for QuantiFERON® TB Gold In-tube (Cellestis, Victoria, Australia) (QFT) and a tuberculin skin test (TST) was administered at baseline. Those with previous or current TB or with a previous severe reaction to TST did not have a TST performed, in order to prevent severe allergic reactions. The reading of the tuberculin skin test took place between 48 and 96 hours after administration, slightly longer than the more commonly used limit of 72 hours but there are data and recommendations which suggest that this is acceptable [16], [17]. A trained nurse examined each adolescent for a BCG scar. Participants were screened for TB at baseline (the overall number of cases diagnosed will be reported here but details of these cases are the subject of another publication (4 above)).

All participants enrolled were scheduled for follow up visits after two years which included a blood draw for QFT and the administration of a TST. About half underwent three monthly visits prior to this which included six monthly QFTs and annual TSTs to compare follow up strategies while the other half were seen only at baseline and two year visit (details of the comparison of the two follow up strategies will be reported separately). At follow up visits, those with new symptoms or a new household contact compared to baseline, a converted TST (≥10 mm increase from baseline) or converted QFT (change from negative to positive) were investigated for active TB. In addition, passive surveillance was conducted of TB clinic and hospital admission registers in the area for any TB cases diagnosed between visits. Investigation for TB involved the collection of two sputum samples for smear examination on two separate occasions. For persons with at least one positive smear, a culture was performed, a chest x-ray done and an HIV test offered. The radiologist’s report on the chest –x ray was used to classify chest-x-ray findings.

### Study Duration

Enrolment started in July 2005 and was completed in April 2007. Follow up was completed at the end of February 2009. Owing to financial constraints, about 10% of the two year visits were performed one to two months short of two years towards the end of the study. Follow up was thus continued for a minimum of 22 months. Those completing their two year visits were followed up passively until all other subjects had completed their two year visits. This gave a maximum follow up time of 3.8 years.

### Definitions

The protocol definition of a TB case was a diagnosis of intrathoracic tuberculosis with either two positive sputum smears and/or one single positive sputum culture (“bacteriologically confirmed TB”). However, data on all individuals placed on TB treatment by a physician were recorded (“all TB”). A chest x-ray consistent with active tuberculosis was defined as “compatible with TB” - this included pleural effusions. An “abnormal chest x-ray” was defined as any abnormality judged to be evidence of active disease including TB and evidence of old/previous disease.

### Sample Size Determination

Those agreeing to participate determined the sample size. Based on routine TB programme data, we expected to find an incidence rate of bacteriologically confirmed TB of 0.5 per 100 person years. With an anticipated sample size of 6,500 and an expected incidence rate of bacteriologically confirmed TB of 0.5 per 100 person years over two years of follow up, we expected to yield a 95% confidence interval (precision) of approximately 0.4 to 0.6/100 person years.

### Data Analysis and Statistical Considerations

Data were captured in a Microsoft Access database, and analysed with STATA version 11.0 (Statacorp, Texas, USA). Data were verified and validated prior, during and after data entry according to a data entry standard operating procedure.

Total person-time for TB incidence analysis was calculated from date of enrolment to date of the last visit of the last participant, TB diagnosis or death, whichever occurred first. Those lost to follow-up were assigned the duration of time to when last seen plus half the duration between that visit and the next missed visit. Univariate analysis was performed on demographic, socio-economic and clinical characteristics examining their association with “bacteriologically confirmed TB”. Hazard rates with 95% confidence intervals were calculated using poisson regression. The design effect was accounted for during statistical analysis, the clusters being the 11 schools from which the participants were enrolled. Kappa statistics were used to evaluate collinearity amongst the potential risk factor variables to avoid over-matching in building models for multivariate analysis. The risk factors for TB disease were analysed in a multivariate Cox regression model using the statistically significant variables (p<0.05) on univariate analysis to determine adjusted hazard ratios.

### Ethics

This study was approved by the Faculty of Health Sciences Human Research Ethics Committee of the University of Cape Town. Written informed consent was obtained from the parents of adolescents and assent obtained from adolescents.

## Results

6,363 adolescents were enrolled (58% of 10,492 registered at local schools and 50% of the estimated total adolescent population of 12,641 in the study area). Enrolment by school ranged from 22.2% to 74.4%. 5203 (81.8%) participants completed their two year visits, 660 (10.4%) withdrew, 11 (0.2%) died and 489 (7.7%) were lost to follow up prior to their two year visit. 84% (1,939/2,310) of participants who met the criteria for investigation underwent the required tests.

### Participant Baseline Profile

The demographic and clinical profile of participants at enrolment is shown in Table 1. There was a slight predominance of females (54%) in the study population compared to the source population data supplied by the Department of Education (50% were female). There was a greater proportion of younger adolescents (≤15 years) than those >15 years since there were fewer adolescents in the higher school grades. BCG scars were faded in some adolescents so it was difficult to be sure about their presence in certain instances. Twenty one cases of TB were diagnosed at baseline of whom 19 were culture positive.

**Table 1:** Unadjusted analysis of risk factors for TB disease for bacteriologically confirmed TB cases.

N = 6,363	N Participants (%)	No of TB cases	Hazard rate/100 pyrs*	Hazard ratio	95% CI**#
**Gender**					
Female	3,458 (54.3%)	45	0.55	1.7	0.5–5.3
Male (ref***)	2,905 (45.7%)	22	0.33	1.0	-
**Age (years)**					
>15	2,760 (43.4%)	31	0.49	1.2	0.7–2.0
≤15 (ref)	3,603 (56.6%)	36	0.42	1.0	-
**Racial group**					
Black and mixed race	5,921 (93.1%)	67	0.48	Perfect predictor	
Indian/white (ref)	442 (6.9%)	0	0.00		
**Parent income: classified on at least one parent’s income**					
<R4000/month	5,313 (83.5%)	62	0.50	2.3	0.6–8.7
>R4000/month (ref)	1,050 (16.5%)	5	0.22	1.0	–
**Maternal highest education level**					
Primary school or less	1,893 (29.8%)	28	0.63	1.7	1.1–2.7
High school or more (ref)	3,482 (54.7%)	24	0.29	1.0	–
Unknown	988 (15.5%)	15	0.68	1.6	1.1–2.4
**Paternal highest education level**					
Primary school or less	849 (13.3%)	12	0.59	1.4	0.6–3.0
High school or more(ref)	2,042 (32.1%)	15	0.31	1.0	–
Unknown:	3,472 (54.6%)	40	0.50	1.3	0.8–2.0
**BCG scar**					
Absent	1,813 (28.5%)	24	0.59	1.5	1.0–2.2
Present (ref)	2,565 (40.3%)	22	0.36	1.0	–
Unknown (Not sure)	1,985 (31.2%)	21	0.46	1.0	0.6–1.6
**Current or prior TB household contact**					
Yes	1,728 (27.2%)	31	0.77	2.3	1.1–4.8
No (ref)	4,635 (72.8%)	36	0.45	1.0	–
**Previous TB**					
Yes	639 (10.0%)	14	0.93	1.5	0.7–3.2
No (ref)	5,709 (89.7%)	73	0.55	1.0	–
Unknown	15 (0.3%)	0	0.00	0	
**TST status at baseline**					
TST positive (≥5 mm)	3,115 (49.0%)	44	0.61	3.0	1.4–6.5
TST negative (ref)	2,456 (38.6%)	12	0.20	1.0	–
TST unknown****	702 (12.4%)	11	0.68	1.6	0.7–3.7
**QFT status at baseline**					
QFT positive	3,233 (50.8%)	48	0.65	2.4	1.4–4.2
QFT Negative (ref)	2,804 (44.1%)	17	0.27	1.0	–
QFT indeterminate or unknown	326 (5.1%)	2	0.21	0.5	0.05–4.7

* pyrs – person years.

** 95% CI = 95% confidence interval.

#adjusted for the design effect.

*** ref – reference.

**** Those with previous or current TB or with a previous severe reaction to TST did not have a TST performed, in order to prevent severe allergic reactions.

### Profile of Incident Cases

There were 87 TB incident cases detected during follow up, 67 (77.0%) of whom met the protocol definition (bacteriologically confirmed TB). A profile of the cases is set out in Table 2. Most incident cases (61 or 70%) were culture positive and most (63 or 72%) had chest –x-ray changes suggestive of tuberculosis. All those diagnosed with TB (87) were offered HIV testing, 61 (70.1%) of whom accepted and of whom only one (1.6%) was found to be positive.

**Table 2:** Profile of TB incident cases.

Variable	Categories	All casesNumber (%)	Bacteriologically confirmed casesNumber (%)
Number of cases	Total	87 (100%)	67 (100%)
Site of disease	Intrathoracic Extra-thoracic	84 (97%) 3 (3%)	67 (100%) 0 (0%)
Smear and culture results	Smear and culture positive Smear negative, culture positive	42 (48%) 19 (22%)	42 (63%) 15 (22%)
	Smear positive, culture negative	9 (10%)	8 (12%)
	Smear and culture negative	13 (15%)	0 (0%)
	Smear positive, culture not done	2 (2%)	2 (3%)
	Smear negative, culture not done	2 (2%)	0 (0%)
Chest x-rays	Compatible with TB:	63 (72%)	51 (76%)
	Abnormal but not typical of TB	8 (9%)	6 (9%)
	Compatible with old/healed TB	5 (6%)	4 (6%)
	Normal	3 (3%)	1 (1%)
	Not done/missing/no report	8 (9%)	5 (7%)

### Incidence Rates

Incidence rates using varying definitions of what constitutes a TB case are set out in Table 3. This resulted in a range of incidence rates from 0.30 to 0.59/100 person years. The highest annual number of cases occurred during the second year of follow up i.e. 33 (49%) of bacteriologically confirmed cases (Table 4). Ten additional cases (15%) were detected through surveillance after the two year visit. The rates of disease in the successive years of follow up were not statistically significantly different from each other. Incidence rates varied widely amongst the different schools from 0 to 2% cumulative incidence and 0 to 0.9/100 person years incidence rates suggesting the possibility of micro-epidemics in certain schools but confidence intervals were wide making these data difficult to interpret. Eight bacteriologically confirmed cases were diagnosed within 6 months of enrolment. If we consider the possibility that these may have been cases missed at baseline and were in fact prevalent cases, then the incidence rate based on the bacteriologically confirmed cases drops to 0.40 per 100 person years (pyrs) (95% CI 0.27–0.58). Incidence rates by TST induration size were as follows: <5 mm–0.20/100 pyrs (95% CI 0.11–0.38), 5–9 mm–0.17/100 pyrs (95% CI 0.04–0.71), 10–14 mm–0.40/100 pyrs (95% CI 0.30–0.53) and ≥15 mm–1.18/100 pyrs (95% CI 0.77–1.81). There was thus a trend of increasing incidence with increasing induration with an induration of ≥15 mm being significantly higher than the other categories.

**Table 3:** TB incidence rates using different case definitions.

	Cases	Cumulative incidence (%) (Mean 2.3 years of follow up) N = 6,363	Incidence rate (per 100 pyrs*) (95% CI**)***. pyrs = 14,786
All	87	1.36	0.59 (0.36–0.96)
Bacteriologically confirmed	67	1.05	0.45 (0.29–0.72)
Culture confirmed	61	0.96	0.41 (0.23–0.71)
Chest x-ray compatible with TB	63	0.99	0.43 (0.27–0.68)
Culture positive and chest x-ray compatible with TB	45	0.71	0.30 (0.17–0.54)
Culture positive and abnormal chest x-ray(includes those compatible with TB)	54	0.85	0.37 (0.21–0.62)
Bacteriologically confirmed TB and chest x-ray compatible with TB	51	0.80	0.34 (0.21–0.56)
Bacteriologically confirmed TB and abnormal chest x-ray	61	0.96	0.41 (0.27–0.64)

* pyrs = person years.

** 95% CI = 95% confidence interval.

*** adjusted for the design effect.

**Table 4:** Annual incidence rate by year of study up to February 2009 for bacteriologically confirmed TB* (n = 6,363).

Year	Persons	Cases	Pyrs	Incidence rate (per 100 pyrs**)(95% CI***)****
Yr 1	6363	23	6,245	0.37 (0.19–0.72)
Yr 2	5784	33	5,557	0.59 (0.40–0.88)
Yr 3	4884	11	2,762	0.40 (0.21–0.75)
Total		67	14,786	0.45(0.29–0.72)

* No cases were reported in year 4 of follow up so this rate has not been included in the table.

** pyrs = person years.

*** 95% CI = 95% confidence interval.

**** adjusted for the design effect.

### Risk Factors for TB

An unadjusted univariate analysis of the relationship between demographic, socio-economic and clinical variables, and TB incidence using hazard ratios is set out in Table 1. A comparison between those having three monthly follow up versus those with just a baseline and two year visit showed no statistically significant difference in incidence rates and is not shown in this analysis. In unadjusted analysis, statistically significant risk factors for bacteriologically confirmed TB were being of black or mixed race origin compared to being of white or Indian origin, maternal education primary school or less and maternal education unknown, current or prior household contact, a positive TST and a positive QFT. Absence of a BCG scar was of borderline statistical significance. These risk factors were considered for fitting in a Cox regression model. We could not include ethnic group since there were no cases in the reference group. Maternal education primary school or less and maternal education unknown were combined into a single variable. BCG coverage in South Africa is high at more than 95%. We therefore assumed that those with an “unknown/unsure” scar were BCG vaccinated and these were grouped with those with a definite scar present for the Cox regression analysis. The following comparisons were tested for collinearity using the kappa statistic: TST versus QFT, prior or current household TB contact versus TST and prior or current household TB contact versus QFT since all three were potential indicators of TB exposure. Only the TST and QFT outcomes were found to be collinear (percentage agreement 85.0%, kappa 0.7). They could thus not be fitted into a model simultaneously. In adjusted analysis (Table 5), absence of a BCG scar, maternal education primary school or less, or unknown and a positive QFT were statistically significant predictors of TB disease while prior household contact was not. When QFT was replaced with TST in the model (not shown), the TST was also a significant predictor with a hazard ratio of 2.4 (95% CI 1.2–4.8). In this model, maternal education primary school or less or unknown remained a predictor of TB disease (hazard ratio 1.9, 95% CI 1.1–3.1) but not absence of a BCG scar. When we defined the presence of a BCG scar strictly and excluded those with scar “unknown/not sure” and repeated the Cox regression (N = 4061), BCG scar and QFT were both only of borderline significance, hazard ratios 1.7 (95% CI 0.9–3.2, p = 0.06) and 1.7 (95% CI 1.0–2.9, p = 0.07) respectively while maternal education of primary school or less or unknown remained significant at hazard ratio 1.4 (95% CI 1.0–1.9, p = 0.04). With this reduced dataset, when TST replaced QFT in the model, only TST remained significant 2.4 (95% CI 1.3–4.3, p = 0.007).

**Table 5:** Adjusted analyses of risk factors for bacteriologically confirmed cases of TB (N = 6,036).

Variables	Hazard Ratio	P> z	95% CI* #
BCG scar absent	1.5	0.04	(1.0–2.1)
Positive baseline QuantiFERON	2.0	0.02	(1.2–3.3)
Mother’s education primary school or less, or unknown	1.8	0.01	(1.2–2.7)
Prior or current household TB contact	1.7	0.15	(0.8–3.9)

* 95% CI = 95% confidence interval.

# adjusted for the design effect.

## Discussion

We found a TB incidence rate of 0.45/100 person years of TB in school-going adolescents in a high burden area. The clinical profile of TB cases was mainly of the adult type: most were confirmed bacteriologically and most had evidence of chest x-ray abnormalities. Significant predictors of TB disease during follow up included demographic, socio-economic and latent TB infection variables. While it was notable that most incident cases occurred in the second year of follow up, differences across the years were not statistically significant.

The British MRC trial in adolescents aged 14.5–15.5 years conducted from 1950–1970 showed an annual incidence rate of 0.25 per 100 in the first five years of the study in the BCG unvaccinated group who were TST negative at enrolment with a similar rate in those with a TST of >15 mm at enrolment [5]. This rate was similar to the 0.2 per 100 found in the TST negative group in our study but not to those with an induration of ≥15 mm who had a significantly higher incidence rate than those with a negative TST. One important difference was that the adolescents in our study had mostly been vaccinated with BCG at birth. Those who were BCG vaccinated in the MRC trial had a lower rate of 0.04 per 100 but had been vaccinated in adolescence as part of the trial so cannot be compared to the adolescents in our study. Children who were aged 7–14 years of age in the Brazilian BCG revaccination trial had rates of 0.03 per 100 person years [4], much lower than in our study. This is probably due to the difference in burden of TB in each country given that South Africa (981/100,000) has a much higher overall incidence rate of TB than Brazil (85/100,000) [1]. The TB incidence rate found in this study is similar to that based on routine TB control programme data in our study area. It was also similar but not identical to the rate reported in a prior publication on this cohort (0.43/100 person years) due to that analysis focusing on a subset of the cohort (5244) where participants with both baseline TST and QFT results were required for analysis [14].

The profile of cases in our study was typical of adult type disease, a finding similar to that shown in a different adolescent population in South Africa by Weber et al [6]. They reviewed the TB diagnosis records of 324 adolescents aged 10–18 years and found 78% to have had bacteriological evidence of disease and 94%, intra-thoracic lesions on chest x-ray. They found 10% with evidence of primary TB whereas we found none, but their lower age limit was younger (10 years) than in our study (12 years). Very few extra-thoracic cases of TB were detected in our study similar to the 6% of cases reported as extra-thoracic by Weber et al [6]. At least 87% of cases in our study had abnormal chest x-rays confirming the microbiological findings of evidence of TB disease.

The choice of TB case definition resulted in some variation in the TB incidence calculation. Given the need for a specific definition in a clinical trial setting to avoid undermining efficacy estimates [12], [13], careful consideration needs to be given to the endpoint case definition for clinical trials. Unlike the pediatric setting where the disease is pauci-bacillary and where reaching consensus on endpoints for infant trials has been difficult [18], most cases in adolescents were microbiologically confirmed suggesting that the use of a highly specific definition is feasible.

We have shown previously that latent TB infection as demonstrated by a positive TST or QFT is associated with a higher risk of progressing to TB disease [14]. The analysis in this paper shows specifically that this relationship persists even when confounders are controlled for. The treatment of latent TB infection with isoniazid as prophylaxis (IPT) is not standard of care in South Africa for HIV negative persons over the age of 5. Fifty percent of adolescents as shown in our study and a higher proportion of adults are latently infected [19]. Implementation of IPT in South Africa would thus be very resource intensive. The benefits of IPT in high TB burden settings for HIV negative adolescents and adults are not clear. A recent large study of IPT in miners in South Africa showed benefit in reducing the risk of TB only while isoniazid was being taken but this benefit was lost when treatment was completed [20]. The university ethics committee which approved this study did not require the provision of INH prophylaxis in adolescents who were latently infected.

The fact that the absence of a BCG scar was shown to be linked to a higher risk of developing TB is surprising given the high population burden of TB in a country with a high coverage of BCG (>95%) [21]. The benefit was only found when QFT was part of the multivariate model but not when it was replaced with TST nor when the presence of the BCG scar was strictly classified and those whose scars were classified as “unsure” were excluded from analysis. There should therefore be caution in interpreting this finding and the absence of a BCG scar may not be indicative of an absent immune response to BCG or indicative of not receiving BCG. A study in India showed no difference in leukocyte migration levels in young BCG vaccinated children between those who developed a BCG scar and those who did not [22]. A recent editorial referencing this and other studies also commented on the lack of correlation between adaptive cellular immune responses and BCG scar formation, and the authors recommend further studies to clarify the differences between those with and those without a BCG scar [23].

The fact that a low or unknown level of maternal education is linked to progression to TB disease is interesting and requires further investigation but surprisingly, parental low income was not, given the known relationship between poverty and TB. Also unexpected was the fact that household TB contact was not shown to be a statistically significant risk factor for TB on adjusted analysis. This may be because the study was not sufficiently powered to show this relationship or due to household contact being very common in this high burden setting. Black or mixed race was a perfect predictor of the onset on TB. It is likely that this association is linked to underlying socio-economic factors given South Africa’s history [24] but this could not be properly tested in multivariate analysis.

The TB incidence rates measured in this study show that large sample sizes will be needed to power clinical trials evaluating the efficacy of new TB vaccines in adolescents. For a vaccine trial expected to show a vaccine efficacy of 60% at a 95% significance level and with 80% power, a study duration of 2 years with completion rate of 82% and cumulative incidence of bacteriologically confirmed TB of 1.05% in the unvaccinated group (as found in this study [Table 3]), a sample size of 6400 participants would be needed in a study with a 1∶1 vaccine to placebo ratio.

This study has a number of limitations: HIV testing was not done routinely because it was believed that this population would not have a high prevalence of HIV. This decision was vindicated by the finding that among participants diagnosed with TB, only one was confirmed to be HIV positive with most agreeing to be tested. Besides the data on gender distribution, no other data was available on those not enrolled in the study. A proportion was older than 18 but there was no exact data on this. In addition, many refused to participate due to fear of blood draws. Our results may thus be biased because of differences between the enrolled and unenrolled populations but we do not believe that this was major given the main reason given for refusal to participate and given that others were not eligible due to their age.

The selections of schools as the source of participants introduced a cluster effect and this produced incidence rates with wider confidence intervals than would have been produced by a purely random sample. While the majority of participants who met the criteria for investigation for TB underwent the required tests, some (371) did not which may have resulted in the actual number of cases being underestimated. However, we believe that the surveillance system would in most instances have eventually picked up cases missed in this way. Hypothetically, given a cumulative incidence of 1.05%, 4 TB cases could have been missed in this group. Smear negative, culture positive TB cases diagnosed early in year one might possibly be cases missed at baseline given that smears were the main method of investigation. In a clinical trial setting using more rigorous screening methods, such cases would have been picked up at baseline and the rates in this study may therefore be an overestimate of incidence relative to what might be expected in a clinical trial. An endpoint committee was not used for case classifications. The case classifications were purely data driven with chest x-ray findings being based only on the radiologists reports. Given that most data items were clear-cut, we do not believe that an endpoint committee would have classified cases significantly differently.

Finally, this study represents the burden of disease at schools only. Adolescents not at school are likely to be located in poorer environments and the TB incidence rate in the total population of adolescents is likely to be higher.

In conclusion, the incidence rates in school-going adolescents in a high burden TB setting ranged from 0.30 to 0.59 per 100 person years using varying case definitions. These data will be essential for the planning of clinical trials of new TB vaccines in adolescents. The risk factors shown to be associated with TB will be useful in estimating TB rates in various risk groups in these clinical trials.
